# The value of CT-based energy imaging to discriminate dominant side lesions in primary aldosteronism

**DOI:** 10.3389/fendo.2023.1121388

**Published:** 2023-04-14

**Authors:** Minggang Huang, Di Yang, Yan Zhang, Yuqing Zhang, Yue Mu

**Affiliations:** Shaanxi Provincial People’s Hospital, Xi’an, Shaanxi, China

**Keywords:** primary aldosteronism, dual-energy computed tomography, adrenal venous sampling (AVS), adrenal adenoma, adrenal hyperplasia

## Abstract

**Purpose:**

The current clinical discrimination of the dominant side of primary aldosteronism (PA) mainly relies on invasive adrenal venous sampling (AVS) examination. This study investigated the feasibility of dual-energy CT energy imaging parameters as a novel biomarker in identifying bilateral adrenal dominant lesions.

**Methods:**

Fifty PA patients with bilateral lesions who underwent CT and AVS of the adrenal glands at Shaanxi Provincial People’s Hospital from October 2019 to June 2021 were retrospectively analyzed. Forty-eight patients had successful bilateral blood collection and two failed right-sided blood collection due to venous variation. Forty patients who were classified based on AVS underwent unilateral adrenalectomy and pathological findings confirmed adenoma in all cases. Quantitative dual-energy CT parameters were measured for all adrenal lesions, and the differences in dual-energy CT energy spectrum imaging parameters between the dominant and nondominant adrenal lesions were compared.

**Results:**

Among forty-eight PA patients with bilateral lesions, forty patients with preoperative AVS-determined lesions on the dominant side underwent unilateral adrenalectomy, and eight patients without the dominant side were treated with medication. The iodine concentration difference (ICD) in the arteriovenous phase was more significant in the 40 cases of primary aldosteronism with dominant adrenal lesions than in the nondominant adrenal lesions (1.18 ± 0.45 vs 0.41 ± 0.42). The NICAP was higher in the dominant adrenal lesions than in the non-dominant lesions (0.39 ± 0.39 vs 0.14 ± 0.05). The sensitivity and specificity of the diagnosis of the dominant adrenal lesion were 88.2% and 82.4% using the ICD of 0.68 as the threshold value.

**Conclusion:**

Conventional CT has lower diagnostic value for dominant adrenal lesions, and CT-based energy imaging can be a new assessment method as a complement to AVS in identifying bilateral dominant adrenal lesions.

## Introduction

Primary aldosteronism (PA) is a prevalent form of secondary hypertension that carries a higher incidence of cardiovascular complications compared to hypertension alone (1), Therefore, accurate diagnosis and treatment of PA are crucial for clinicians. The major subtypes of PA include idiopathic hyperaldosteronism (IHA), aldosterone-producing adenomas (APA), primary adrenal hyperplasia, and familial aldosteronism (2). The diagnosis of PA is important for giving patients the opportunity for specific surgical or pharmacological treatment to reduce cardiovascular risk (1).While Computed Tomography (CT) and adrenal venous sampling (AVS) are commonly used for diagnosing PA subtypes, conventional CT has limited diagnostic value in detecting dominant adrenal lesions (3). Clinical guidelines suggest that AVS performed by an experienced interventionalist is the gold-standard test for differentiating unilateral and bilateral PA (4). However, the invasiveness, high cost, and complexity of operation limit the clinical application of AVS (5). The advent of a new generation of energy CT, which utilizes CT image acquisition and post-processing technology, has enabled functional imaging, including dual-energy computed tomography (DECT) that allows for the clinical diagnosis of adrenal tumors. Despite this, research on DECT and its energy imaging to differentiate dominant adrenal lesions remains scarce.

Therefore, the aim of this study is to investigate the feasibility of utilizing DECT energy imaging as an alternative to AVS in diagnosing bilateral adrenal dominant lesions.

## Method

### Patient cohort

**Figure 1:**
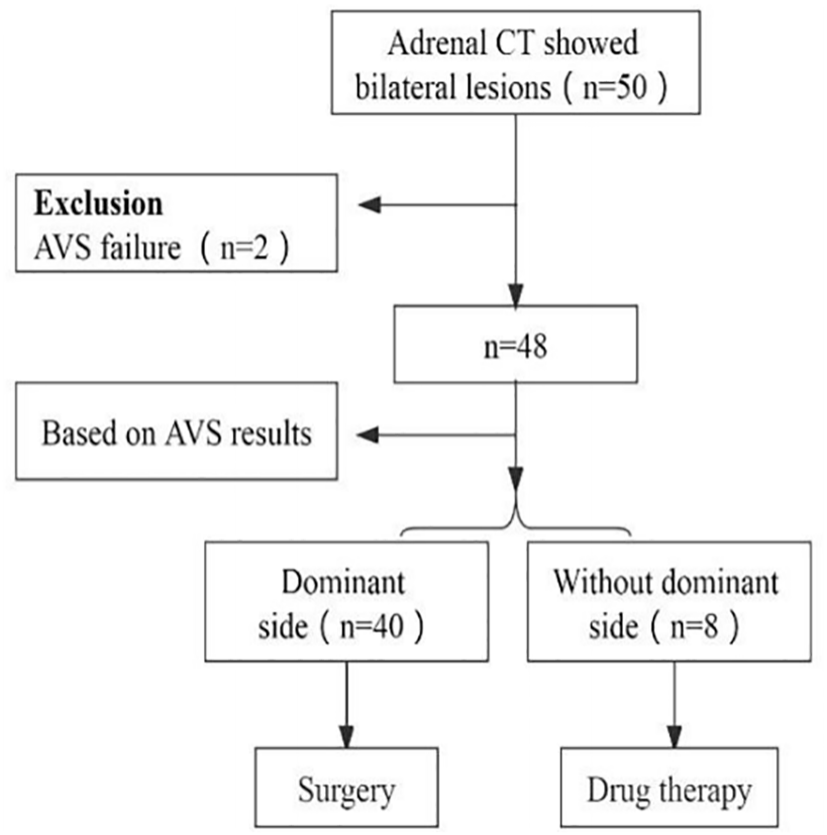
Study cohort.

A total of fifty patients diagnosed with PA, who had bilateral lesions and underwent CT and AVS of the adrenal glands between October 2019 and June 2021 at Shaanxi Provincial People’s Hospital, were included in this retrospective analysis. CT scans showed bilateral adrenal lesions in all patients. Bilateral blood collection was successful in forty-eight patients, while two patients failed to collect right-side blood due to venous variation. Histopathological examination confirmed adenoma in all 40 patients who underwent unilateral adrenalectomy based on AVS classification, whereas eight patients with bilateral PA were managed with medication (refer to **Figure 1**). AVS results revealed that 40 patients had dominant-side lesions, while eight had nondominant-side lesions. Quantitative parameters of dual-energy CT were evaluated for all adrenal lesions. According to the AVS results, the 40 patients with dominant-side lesions were divided into a dominant-side group and a nondominant-side group, and the measurements were obtained accordingly. The clinical and biochemical characteristics as well as quantitative dual-energy CT parameters of both groups were evaluated. Prior to screening, all patients were assessed and either 2 weeks after discontinuing the use of all antihypertensive drugs (except calcium channel blockers and α-adrenergic blockers) or 6 weeks after discontinuing spironolactone or eplerenone. Screening for PA was based on the plasma aldosterone concentration (ng/dl) to plasma renin activity (ng/ml/h) ratio (ARR) of >30. Diagnosis of PA was confirmed using captopril provocation or saline infusion tests, as per the guidelines published by the Japanese Society of Hypertension in 2014 (6). Cortisol cosecretion was excluded based on the dexamethasone inhibition test.

### Adrenal examination: CT scanning procedure

All images were acquired with the patient in the supine position using a second-generation Siemens SOMATOM Definition Flash dual-energy computed tomography (DECT) scanner. Following the acquisition of unenhanced CT images, the STELLANT SCT-210 double-barrel high-pressure syringe (MEDRAD, USA) was used to inject 320 mg/mL of iopromide at a rate of 2.5-4 mL/s. Subsequently, 20 mL of saline was injected at the same rate for 25-30 s for the arterial phase scan and 65–70 s for the venous phase scan. Axial slices of 0.6 mm were acquired and transferred to the workstation (Syngo.via) for further analysis.

### Image analysis and data measurement

The acquired data was transferred to the Syngo.via workstation, and the arterial and venous dual-phase dual-energy CT data were transferred to the Dual application. Three independent radiologists performed circumferential region of interest (ROI) measurements on the largest two-dimensional cross-sectional area of the adrenal lesion, using an ROI size of approximately 0.5-1.0 cm2. The ROI measurements were averaged, and the area containing only the lesion was outlined. The vendor-provided “Liver VNC” program was utilized to measure the lesion size, the intravenous iodine concentration, and the homogeneous abdominal aorta. The iodine concentration (IC) of each adrenal lesion in the arterial and venous phases was measured and recorded separately; IC_AP_ was the iodine concentration of arterial phase and IC_VP_ was the iodine concentration of venous phase. Calculate the iodine concentration difference between the arterial and venous phases (ICD=IC_VP_-IC_AP_). The ratio of the iodine concentration in the enhanced lesion to that in the homogeneous abdominal aorta was recorded as the normalized iodine concentration (NIC) (7).

### Techniques and criteria for adrenal vein blood collection

Non-adrenocorticotropic and adrenocorticotropic stimulation of the collected bilateral adrenal vein blood was performed in the catheterization room. The criterion for successful selective cannulation was a selectivity index (SI) of >2 or 3, which is the ratio of the adrenal vein to inferior vena cava cortisol concentration (8–10). The lateralized ratio was calculated as (adrenal vein A/C ratio on the high-value side)/(adrenal vein A/C ratio on the low-value side). If the lateralized ratio is >2 or 4 (11–14), a dominant lesion is considered to be present on the high-value side.

### Statistical analysis

Quantitative data were presented as mean ± standard deviation or median (25th–75th percentile) and were tested for normality. The paired samples t-test was employed for data with a normal distribution, whereas the Mann–Whitney U rank sum test was used for data that did not follow a normal distribution. Receiver operating characteristic (ROC) curve analysis was utilized to evaluate the diagnostic efficiency of the differential indicators and determine the area under the curve (AUC). Values were presented as mean ± SD, and statistical significance was defined as P < 0.05. The statistical software package SPSS 22.0 (IBM, Armonk, NY, USA) was utilized.

### The primary aldosteronism surgery outcome (PASO)

**Figure 2:**
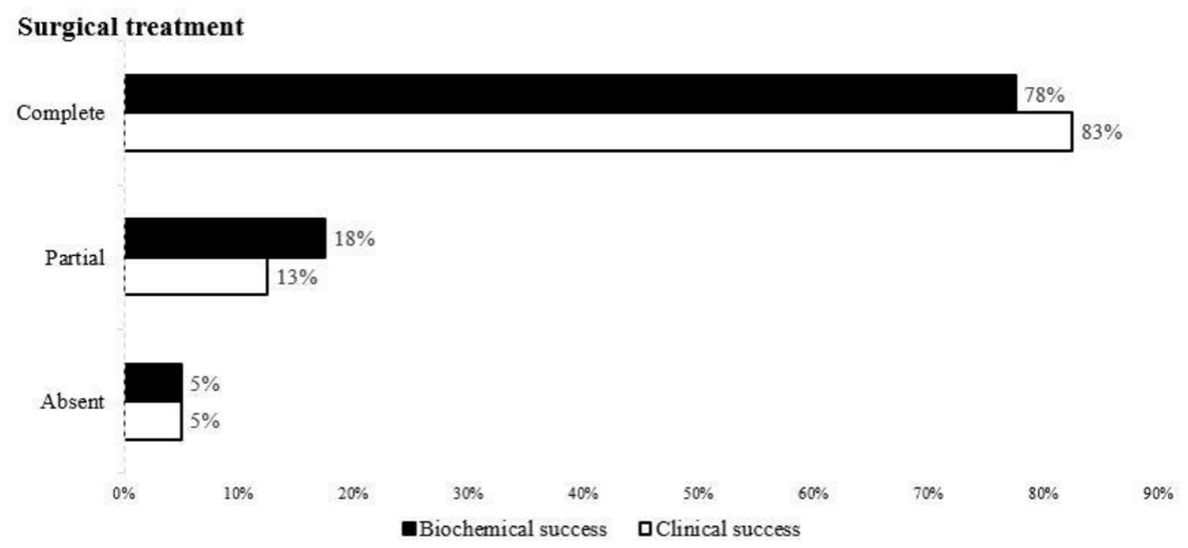
Remission rate of clinical and biochemical findings in all patients who underwent unilateral adrenalectomy according to the PASO study criteria.

Currently, international criteria are in place to assess the surgical outcome of primary aldosteronism (PASO), which involves three categories: (1) complete remission: blood pressure and biochemical parameters (serum potassium, plasma aldosterone and ARR) are normal after stopping antihypertensive drugs after treatment; (2) partial remission: blood pressure decreases to the same level when antihypertensive drugs are required, or is maintained. (3) No remission: no improvement in blood pressure and biochemical parameters, or an increase in the number of drugs required, and it is recommended that the above parameters be evaluated at 6 and 12 months after surgery (15).The outcome categories are illustrated in **Figure 2**.

## Results

**Table 1** displays the demographic and biochemical characteristics of the 40 patients who underwent AVS blood collection. Patients with APA had significantly lower blood potassium levels compared to those with bilateral adrenal hyperplasia (BAH) (2.82 ± 0.44 mg/mL vs. 3.31 ± 0.37 mg/mL, respectively; *P* < 0.05), as well as higher systolic blood pressure and aldosterone secretion (159.80 ± 12.64 vs. 140.75 ± 6.67; 278.95 vs. 155.87, respectively; both *P* < 0.05). Age and renin levels did not differ significantly between the two groups (*P* > 0.05).

**Table 1:** Baseline characteristics of AVS patients.

Projects	APA (n=40)	BAH (n=8)	Total (n=48)	*P* Value
Age	53.83 ± 10.30	56.38 ± 7.69	54.25 ± 9.89	0.511
BMI(kg/m2)	24.37 ± 3.96	24.48 ± 3.06	24.39 ± 3.80	0.945
SBP(mmHg)	159.80 ± 12.64	140.75 ± 6.67	156.63 ± 13.81	0.000*
DBP(mmHg)	105.25 ± 8.30	102.50 ± 10.35	104.79 ± 8.62	0.416
SP(mmol/L)	2.82 ± 0.44	3.31 ± 0.37	2.90 ± 0.46	0.006*
PAC(pg/mL, interquartile)	278.95 (254.03,322.52)	155.87 (106.41,235.82)	273.14 (200.30,309.62)	0.000*
PRC(ng/mL/h, interquartile)	3.91 (1.50,6.57)	1.50 (1.27,2.83)	3.50 (1.50,6.57)	0.718
ARR(interquartile)	80.13 (42.69,158.30)	99.40 (42.70,235.64)	72.29 (40.97,156.24)	0.534

Parameters are shown as mean ± standard deviation or median (25^th^ to 75^th^ percentile). The data were analyzed using Paired sample t test or Mann-Whitney’s U-test between the two groups.

APA, aldosterone-producing adenoma; BAH, bilateral adrenal hyperplasia; SBP, systolic blood pressure; DBP, diastolic blood pressure; SP, serum potassium; PAC, plasma aldosterone concentration; PRC, plasma renin concentration; ARR, PAC/PRC.

*indicates statistical significance.

**Figure 3:**
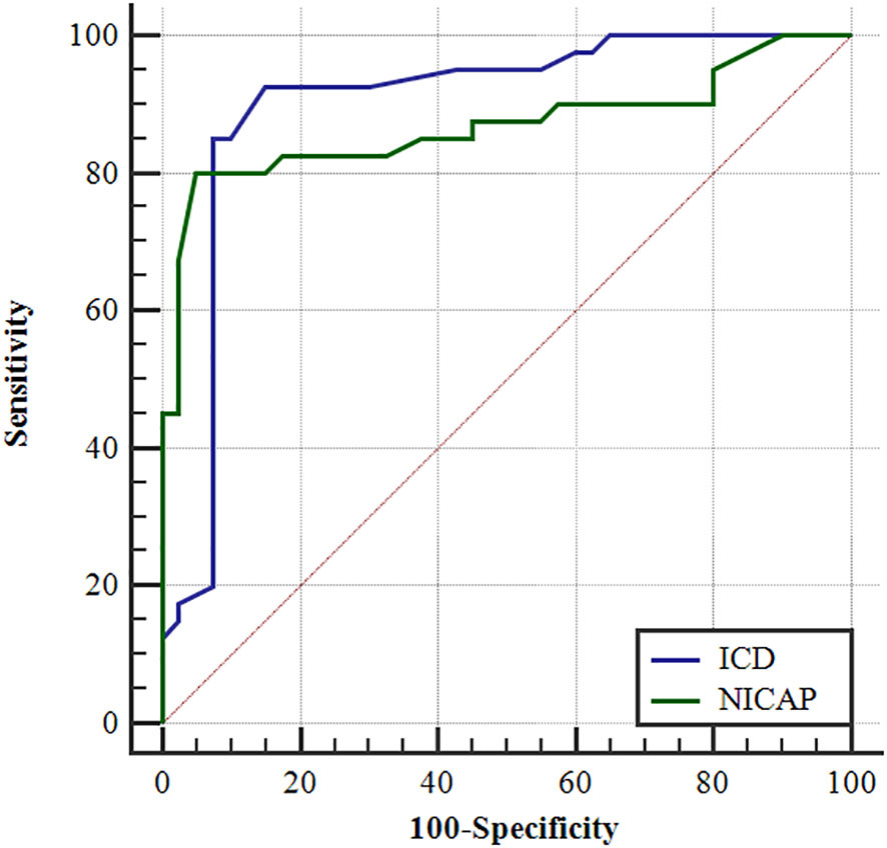
The ROC curves of the ICD and the NIC_AP_ to identify the dominant and the non-dominant side.

In **Table 2**, ICD and NIC_AP_ levels were significantly higher in the dominant-side group compared to the nondominant-side group (1.18 ± 0.45 mg/mL vs. 0.42 ± 0.42 mg/mL; 0.39 ± 0.39 vs. 0.14 ± 0.05, respectively; *P* < 0.05). There were no statistically significant differences in IC_AP_, IC_VP_, and NIC_VP_ between both sides (*P* > 0.05). The AUC for ICD and NIC_AP_ were 0.900 and 0.869, respectively. When the cutoff values for ICD and NIC_AP_ were set as 0.68 mg/mL and 0.199, respectively, both parameters had the best diagnostic efficacy for dominant adrenal lesions, with sensitivities of 92.5% and 85.0% and specificities of 80.0% and 95.0%, respectively (**Figure 3** and **Table 3**). The IC_AP_, IC_VP_, NIC_AP_, NIC_VP_, and ICD of adrenal lesions on both sides in eight patients with idiopathic aldosteronism were similar, with no significant differences (*P* > 0.05) (**Table 4**).

**Table 2:** Comparison of energy parameters on the dominant and non-dominant side of the iodine distribution map.

Groups	IC_AP_ (mg/ml)	IC_VP_ (mg/ml)	ICD (mg/ml)	NIC_AP_	NIC_VP_
Dominant side	2.26 ± 1.06	1.46 ± 0.70	1.18 ± 0.45	0.39 ± 0.39	0.17 ± 0.12
Non-dominant side	2.09 ± 0.53	1.69 ± 0.55	0.41 ± 0.42	0.14 ± 0.05	0.15 ± 0.07
*t* Value	0.917	-1.584	3.989	3.990	0.748
*P* Value	0.362	0.117	0.000*	0.000*	0.457

Parameters are shown as mean ± standard deviation. The data were analyzed using Paired sample t test between the two groups.

IC_AP_, iodine concentration of arterial phase; IC_VP_, iodine concentration of venous phase; ICD, IC_AP_- IC_VP_; NIC_AP_, normalization iodine concentration of arterial phase; NIC_VP_, normalization iodine concentration of venous phase.

*indicates statistical significance.

**Table 3:** Iodine difference and NIC_AP_ to identify the results of ROC curves on the dominant and non-dominant side.

	Cut-off Value	Sensitivity	Specificity	AUC
ICD	0.68	92.6%	85.0%	0.900
NIC_AP_	0.199	80.0%	95.0%	0.869

**Table 4:** Comparison of energy parameters on adrenal iodine maps between the right and left sides in 8 patients without the dominant side.

Groups	IC_AP_(mg/ml)	IC_VP_(mg/ml)	ICD(mg/ml)	NIC_AP_	NIC_VP_
Left sides	2.34 ± 0.84	2.08 ± 0.63	0.24 ± 0.09	0.30 ± 0.10	0.39 ± 0.12
Right sides	2.10 ± 0.68	1.89 ± 0.57	0.19 ± 0.11	0.39 ± 0.12	0.34 ± 0.13
*t* Value	0.575	0.621	0.974	-1.660	0.780
*P* Value	0.893	0.852	0.614	0.338	0.614

Parameters are shown as mean ± standard deviation. The data were analyzed using Paired sample t test between the two groups.

**Figure 4:**
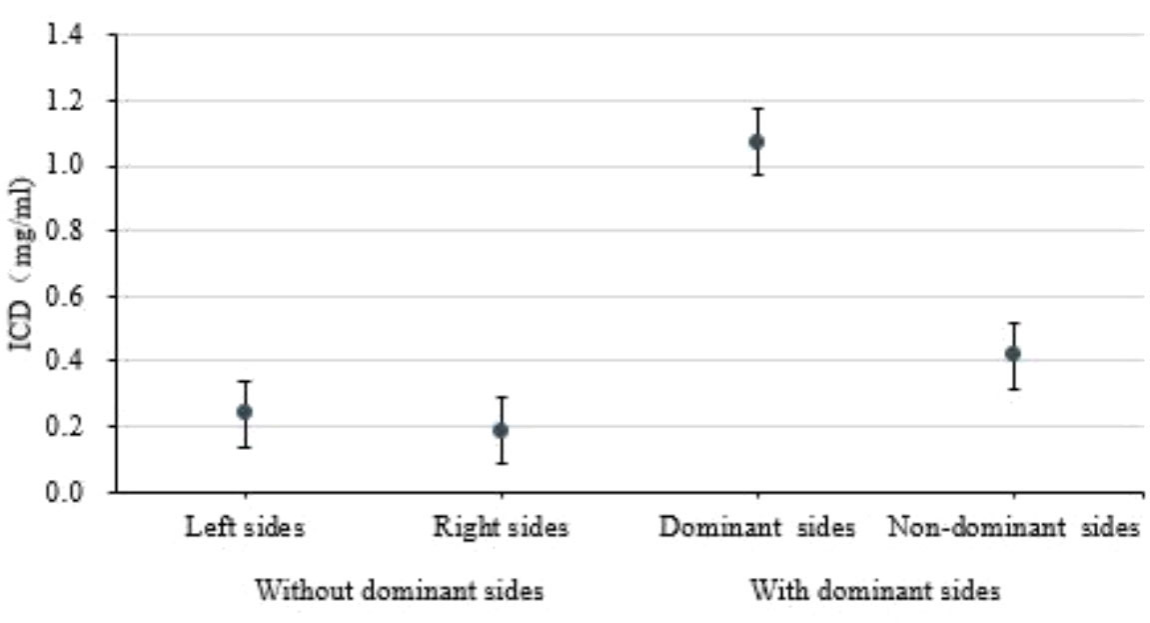
Comparions on both sides in patients with and without dominant side.

**Figure 5:**
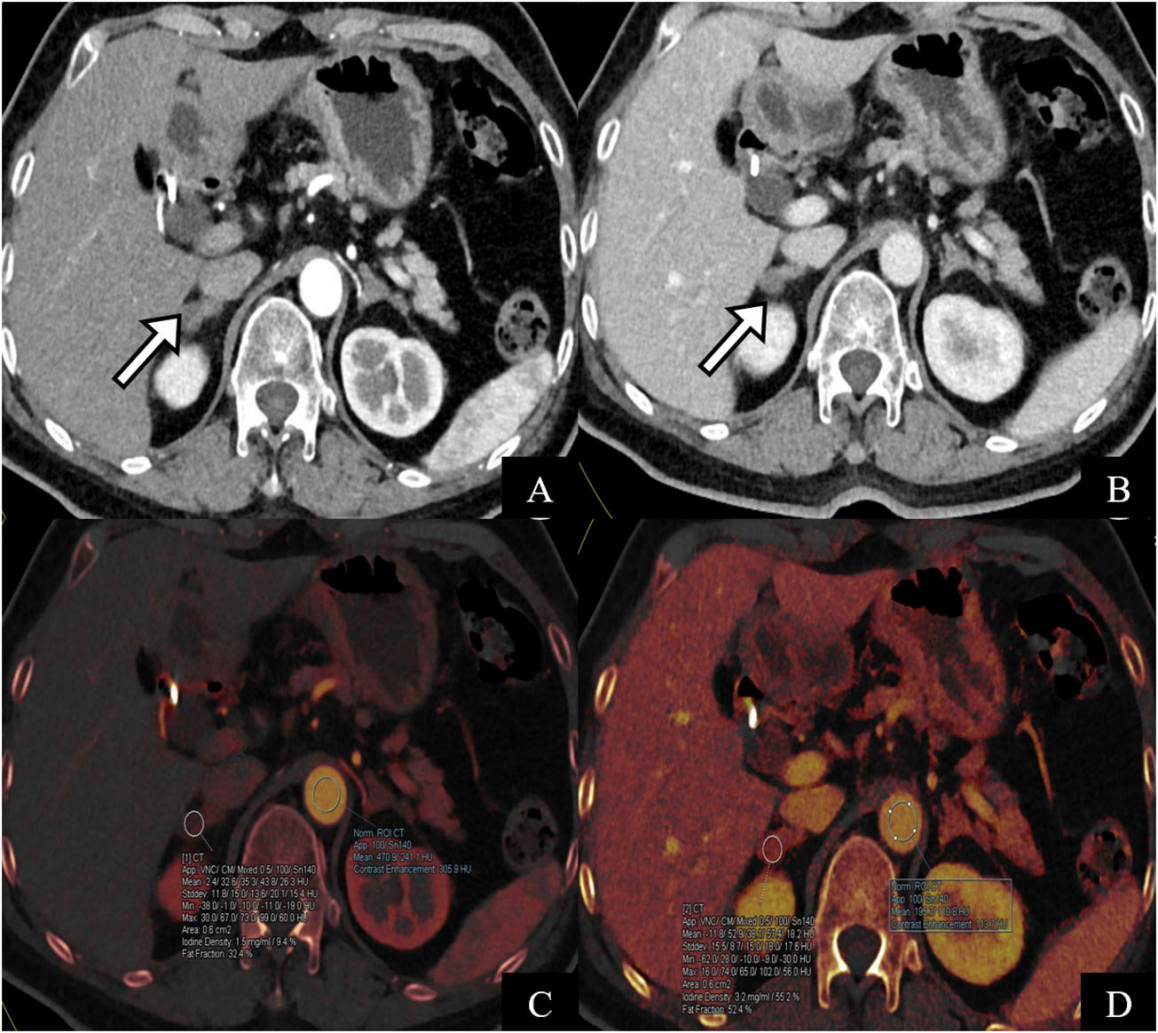
A 64-year-old female patient with surgically pathologically confirmed of primary hyperaldosteronism. The CT scan of the adrenal glands showed soft tissue density of nodular adrenal glands bilaterally **(A, B)** pointed by white arrow). AVS indicated high secretion of aldosterone on the right side. **(C, D)** are iodine maps of the arterial phase. The iodine concentration in the arterial and venous phases was 1.7 mg/ml and 0.61 mg/ml, respectively, and the ICD was 1.09 mg/ml, which is consistent with the diagnosis of the right dominant side of AVS.

As demonstrated in **Figure 4**, the difference in iodine concentration in the arteriovenous phase was higher in the dominant-side group than in the nondominant-side group or the group without a dominant side of the adrenal lesion (*P* < 0.05). In addition, the ICD between adrenal lesions on both sides in the nondominant group overlapped, with no significant difference (*P* > 0.05). Example cases are shown in **Figure 5**.

**Figure 6:**
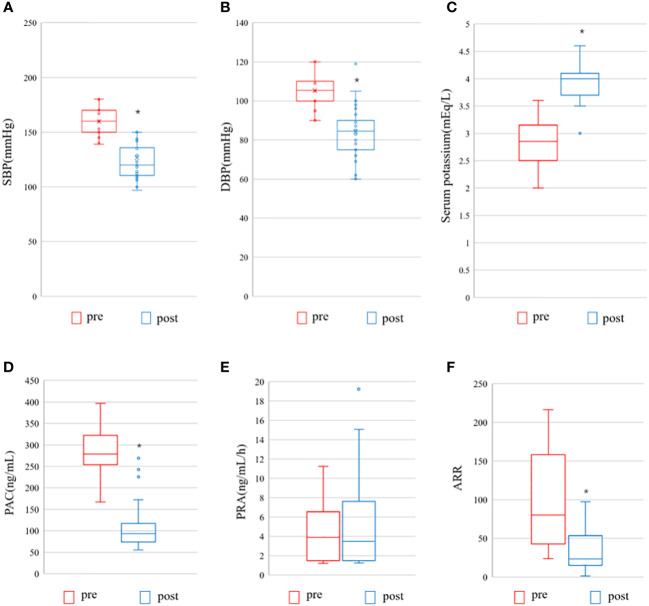
Pre- and postoperative evaluation of the AVS- unilateral primary aldosteronism patients undergoing surgery. Pre, pre-operation; post, post-operation; **(A)** SBP, systolic blood pressure; **(B)** DBP, diastolic blood pressure; **(C)** Serum potassium; **(D)** PAC, plasma aldosterone concentration; **(E)** PRA, Plasma renin activity; **(F)** ARR, Aldosterone-to-renin ration. The data are shown as box plots, were analyzed using the Wilcoxon signed-rank test. *indicates statistical significance.

We also compared preoperative and postoperative clinical and biochemical characteristics of patients with AVS-unilateral PA. A total of 40 patients underwent unilateral adrenalectomy, as displayed in **Figure 6**. Preoperatively, there were no differences in systolic and diastolic blood pressure, number of antihypertensive drugs, plasma renin activity, and aldosterone-to-renin ratio between the two groups. Postoperatively, patients had significantly lower systolic blood pressure, diastolic blood pressure, aldosterone concentration, and ARR than before surgery (*P* < 0.0001), significantly higher serum potassium levels than before surgery (*P* < 0.0001), and no significant difference in renin before and after surgery (*P* = 0.106) (see **Figure 6**).

Finally, the remission rates of clinical and biochemical outcomes of all patients with AVS treated surgically were evaluated according to the criteria of the PASO study (15) (**Figure 2**). The clinical complete success rate for surgical treatment was 82.5% (33/40), the partial success rate was 12.5% (5/40), and failure rate was 5% (2/40). The biochemical complete success rate was 77.5%.

## Discussion

The staging of unilateral primary aldosteronism (PA) is a crucial and challenging step, which can be treated with unilateral adrenalectomy. Currently, adrenal venous sampling (AVS) is recognized as the international gold standard for the diagnosis of PA. The correct localization of AVS and cannulation are key to success; however, the variability and specificity of the anatomical location of the adrenal vein (especially on the right side) and the tendency of the vein wall to collapse have led to a high failure rate of cannulation, approximately 19%-74% (16). Furthermore, a few patients experienced complications such as adrenal hematoma, and the high cost of the procedure has limited the widespread clinical use of this technique (17, 18). CT is the most commonly used imaging method for the adrenal gland because of its fast scanning speed, simplicity, and high spatial resolution. However, CT often inaccurately diagnoses PA subtypes, and the sensitivity and specificity of CT for unilateral lesions (78% and 75%, respectively) are lower than those of AVS (95% and 100%, respectively) (19). Several retrospective studies have reported compliance rates ranging from 31% to 54% for CT and AVS, respectively (2, 18, 20). However, the diagnostic role of DECT energy imaging has not been explored comprehensively. Hence, this study aimed to develop new feasible diagnostic methods for differentiating adrenal CT subtypes.

Immunohistochemical detection of vascular endothelial growth factor (VEGF) expression and microvessel density (MVD) is the gold standard for evaluating tumor angiogenesis. In previous studies, VEGF expression levels were found to be higher in aldosterone-producing adenoma (APA) than in normal adrenal tissue, Cushing’s adenoma, and non-functional adenomas. The microvessel density (MVD) of such examples was found to have a positive correlation with aldosterone, but not with steroids and adrenocorticotropic hormone, which suggests a relationship between adrenocortical tumors and their functional status (21). It is postulated that the strength of secretory function correlates with the level of blood supply in aldosterone adenoma. DECT energy imaging utilizes the distinct attenuation coefficients of different substances at various energy levels to achieve their separation and synthesis (22). These coefficients respond directly to the iodine concentration within the lesion (23), which accurately reflects the blood supply to the lesion. Therefore, we hypothesized that the application of energy CT could indirectly reflect the side of the bilateral adrenal lesions with high secretion and abundant blood supply. The study’s results demonstrated that the iodine concentration difference (ICD) in patients with dominant adrenal lesions was higher than that in patients with non-dominant adrenal lesions (1.18 ± 0.45 mg/mL vs 0.41 ± 0.42 mg/mL, respectively).

Furthermore, conventional CT readings are inadequate in determining functional adrenal changes. Therefore, the iodine concentration may serve as a sensitive quantitative indicator to evaluate the dominant side of the lesion and improve subtype differentiation results. When using a threshold value of 0.68 mg/mL to diagnose dominant adrenal lesions, the sensitivity and specificity were found to be 92.6% and 85.0%, respectively. In this study, eight cases of adrenal CT showed bilateral lesions, but AVS results were not dominant on either side, leading to a clinical diagnosis of idiopathic aldosteronism. The metabolic differences between the two sides, as measured by the iodine concentration in DECT energy imaging, were less than 0.3 mg/mL. These differences were not substantial enough to cause an excessive increase in aldosterone on one side, resulting in an aldosterone-to-cortisol ratio of ≥2 or 4 on both sides.

The iodine concentration in enhanced CT is primarily influenced by the amount of contrast agent injected and the time interval needed to reach the lesion, which is related to the patient’s body weight and blood flow rate. Thus, the concept of “normalized iodine concentration” was introduced to eliminate the influence of extraneous factors. This study further found that the NIC_AP_ of the dominant adrenal lesion was higher than that of the nondominant lesion, leading to better diagnostic efficacy. These findings need to be validated in a larger prospective study.

There are limitations of this study. Firstly, the method was not applicable to all PA patients in this study, but only to patients with bilateral adrenal lesions. Secondly The number of cases present in this study is relatively small and not all patients underwent immunohistochemistry-related studies, and more cases need to be included in the future to validate the results. Thirdly, the inability to measure lesions that cannot be visualized *via* CT necessitates further improvements in this technique. Fourth, this study focuses on the population with aldosterone hypersecretion, but the incidence of patients with primary aldosteronism combined with autonomic cortisol has also gradually increased in recent years, which will also be included in the next relevant studies. The final point is a number of patients with unilateral disease are cases of bilateral but asymmetric aldosterone production with AVS results showing high lateral, these cases should benefit from adrenalectomy and are therefore considered to be patients with unilateral or lateral PA, relevant cases were not included in this study and will be further studied in the future by expanding the study (5). Burrello (24) used supervised machine learning algorithms and regression models to develop and validate two predictive models and a simple 19-point scoring system that stratified patients according to their subtype diagnosis, correctly classifying the majority of patients at both training and validation (accuracy: 82.9-95.7%) and avoiding potential duplication of 77.2% of AVS procedures. In the future, AI-based predictive models may become powerful predictive tools for the diagnosis of PA subtypes, thus potentially reducing the need for at least some CT or AVS and assisting clinical decision making, which is the next direction of our research. Current studies also support the use of aldosterone index (AI) in predicting AVS selectivity and lateralization in patients with PA. AI was studied as selectivity index (ASI) and lateralization index (ALI) by Parasiliti (1) et al. both provided satisfactory accuracy and were also applicable to centers without experienced interventional radiologists.

In conclusion, we found CT-based energy imaging can be a new assessment method in addition to AVS in identifying bilateral dominant adrenal lesions with improved diagnostic accuracy as compared to conventional CT.

## Data availability statement

The original contributions presented in the study are included in the article/supplementary material. Further inquiries can be directed to the corresponding author.

## Author contributions

DY and MH was involved in image data analysis, statistical analysis, and report writing, and was responsible for clinical and imaging data collection, linguistic and grammatical checking of the manuscript, statistical analysis, writing review, and editing. YM, YuZ, and YaZ provided scientific guidance for report writing. All authors contributed to the article and approved the submitted version.
